# Insuline-Like Growth Factor-2 (IGF2) and Hepatocyte Growth Factor (HGF) Promote Lymphomagenesis in p53-null Mice in Tissue-specific and Estrogen-signaling Dependent Manners

**DOI:** 10.7150/jca.60120

**Published:** 2021-08-21

**Authors:** Hsuan-Shun Huang, Sung-Chao Chu, Pao-Chu Chen, Ming-Hsun Lee, Chi-Ya Huang, Hsien-ming Chou, Tang-Yuan Chu

**Affiliations:** 1 Center for Prevention and Therapy of Gynecological Cancers, Department of Research, Buddhist Tzu Chi General Hospital, Hualien 970, Taiwan, ROC.; 2 Department of Hematology and Oncology, Buddhist Tzu Chi General Hospital, Hualien 970, Taiwan, ROC.; 3 Department of Obstetrics & Gynecology, Buddhist Tzu Chi General Hospital, Hualien 970, Taiwan, ROC.; 4 Department of Pathology, Buddhist Tzu Chi General Hospital, Hualien 970, Taiwan, ROC.; 5 School of Medicine, College of Medicine, Tzu Chi University, Hualien 970, Taiwan, ROC.; 6 Department of Life Science, Tzu Chi University, Hualien 970, Taiwan, ROC.

**Keywords:** follicular fluids, lymphoma, *Trp53*, IGF, estrogen

## Abstract

**Background:***Trp53^-/-^* mice are prone to develop lymphomas at old ages. Factors promoting this tumorigenesis are unknown. Here, we showed human ovulatory follicular fluid (FF) largely promotes lymphomagenesis in *Trp53^-/-^*mice at earlier ages. Meanwhile, we clarified that IGF2 and HGF are important cell transforming factors within FF.

**Methods:** To induce tumor formation, 5% FFs, 100 ng/ml IGF2, 20 ng/ml HGF, or both IGF2 and HGF in a volume of 200 µl PBS, was injected into 8-wk-old female *Trp53 ^-/-^* mice at the mammary fat pad. The injection was repeated weekly for up to 7 weeks or extending to 13 weeks to observe the accumulative incidence of lymphomagenesis. Immunohistochemistry staining and gene rearrangement analysis were used to identify the tumor type.

**Results:** By injecting FF into the mammary fat pad weekly, lymphomas developed in 8/16 (50%) of mice by seven weeks. We identified IGF2 and HGF in FF is largely responsible for this activity. The same weekly injection of IGF2, HGF, and their combination induced lymphomas in 4/11 (36%), 3/8 (38%), and 6/9 (67%) mice, respectively. Interestingly, tumorigenesis was induced only when those were injected into the adipose tissues in the mammary gland, but not when injected into non-adipose sites. We also found this tumor-promoting activity is estradiol (E2)-dependent and relies on estrogen receptor (ER) α expression in the adipose stroma. No tumor or only tiny tumor was yielded when the ovaries were resected or when ER is antagonized. Finally, an extension of the weekly FF-injection to 13 weeks did not further increase the lymphomagenesis rate, suggesting an effect on pre-initiated cancer cells.

**Conclusions:** Taken together, the study disclosed a robust tumor-promoting effect of IGF2 and HGF in the p53 loss-initiated lymphomagenesis depending on an adipose microenvironment in the presence of E2. In light of the clarity of this spontaneous tumor promotion model, we provide a new tool for studying p53-mediated lymphomagenesis and suggest that, as a chemoprevention test, this is a practical model to perform.

## Introduction

Lymphoma is a cancer of the lymphatic system corresponding to cells in a lymphocytic lineage that are ontogenetically arrested at various developmental stages. Non-Hodgkin's lymphoma is one of the two main types of lymphoma and comprises the majority (90%) of cases. Non-Hodgkin's lymphomas comprise a range of malignancy with distinct genetic alterations, cellular phenotypes, and clinical presentations. The etiology of these heterogeneous malignancies in most cases remains unknown. A multifactorial panel of risk factors including immunodeficiency, autoimmune 1, chemicals and irradiation, infection 2, and reproductive factors including sex hormones 3, 4 has been identified. These risk factors predispose lymphocytic cells to malignant transformation due to driver mutations. Among them, aberrations of the *TP53* gene is one of the most drivers in the pathogenesis of T cell and B cell lymphomas 5-7. Loss of p53 activity has been reported in diffuse large B-cell lymphoma 8, mantle cell lymphoma 9, intestinal T-cell lymphoma, and peripheral T-cell lymphoma 10. A meta-analysis revealed *TP53* mutation predicts a poorer prognosis of non-Hodgkin' lymphomas 11.

In p53-deficient transgenic mouse models, lymphomas of T cell or B cell lineage are the predominant tumors found at old ages. In the p53 null mice, thymic T-cell lymphoma is the predominant tumor growth 12, whereas *Trp53* missense mutant mice usually grow B-cell lymphomas in the lymph node and spleen 13. These tumors typically develop spontaneously at old age. For instance, in the p53-null mice of C57BL/6 background lymphomas and rare sarcoma developed at 19 ± 4.5 weeks) 12, 14, 15.

In our previous studies, human preovulatory follicular fluids (FFs) collected from *in-vitro* fertilization (IVF) women carry carcinogens such as IGF axis proteins and other growth factors. When directly injected into the mammary fat pad of *Trp53^-/-^* mice, FF could induce the early onset of lymphomas locally and distantly 16. In the later studies, we found these FFs contain a high level of IGF2 17, and HGF (unpublished results) that activates stemness, clonal expansion, and cell transformation 17 of the FF-exposed fallopian tube fimbrial epithelial cells.

From these studies, we have established a rapid lymphomagenic mouse model by direct injection of FF to the mammary fat pad of *Trp53^-/-^* C57BL/6 mice. In this study, we aim to further test the robustness of using IGF2 and HGF to replace FF in this lymphoma mouse model and to investigate the role of microenvironment and estrogen signaling in tumor development.

## Material & Methods

### Follicular fluids

Follicular fluid (FF) aspirates were collected from 16 women (aged 23-47 y) who underwent oocyte retrieval and *in vitro* fertilization program. The follicle sizes were measured before each aspiration by transvaginal sonography by using spectrophotometry, aspirates with blood- or flush medium-contamination were identified and excluded as described earlier 18. Only the yellow color FFs were chosen in experiments. The FF aspirates were centrifuged (1200g, 10 minutes) to remove cell debris and frozen in -80 °C before use. The procurement of clinical specimens for this study was approved by the Institutional Review Board (IRB 106-07-A) of Tzu Chi General Hospital, Hualien, Taiwan. Informed consent was signed by each donor.

### Mouse tumorigenesis studies

The *Trp53^-/-^* mice (STOCK *Trp53^tm1Brd^* Brca1*^tm1Aash^*/J) purchased from Jackson Laboratory (Stock No. 012620) or its background wild type strain C57BL6/J were used for spontaneous tumorigenesis. To induce tumor formation, 5% FFs, 100 ng/ml IGF2, 20 ng/ml HGF, or both IGF2 and HGF in a volume of 200 µl PBS, were injected into 8-wk-old female mice at different sites. *Trp53^-/-^*mice should be chosen with a healthy appearance and without any tumor formed before the experiment. The injection was repeated weekly for up to 7 weeks or extending to 13 weeks to observe the accumulative incidence of lymphomagenesis. Mice were sacrificed at the 8^th^ week or 14^th^ week. A tumor over a centimeter in size would be sacrificed on ethical considerations for experimental animals. The injection sites included the mammary fat pads at the bilateral groin region, subcutaneous of the back skin, and intramuscular at the thigh. For the estrogen-dependence studies, 7-wk-old female mice underwent bilateral ovariectomy. After one week of recovery, they were subjected to the same tumor induction protocol. For estrogen supplement, a micro-osmotic pump (Alzet MODEL 1004) containing 80 nM E2 in a volume of 100 μl was implanted to the dorsal subcutaneous of male *Trp53^-/-^* mice. In a release rate of 0.11μl per hour for 28 days, this would recapitulate the E2 effect as in the female. Alternatively, 80 nM E2 was co-injected with 5% FF in a volume of 200 ul PBS with the same injection protocol mentioned above. For antiestrogen treatment, fulvestrant, 5 mg per mice, was added to FF and subjected to the same injection protocol. For serial transplantation, a tumor piece of 50 mg was cut into small pieces and was transplanted to the mammary fat pat of another 8-wk-old female *Trp53^-/-^* mice. The transplanted tumor was observed after two weeks. All mouse experimental procedures were approved and conducted under the guidelines of the Animal Care and Use Committee of Tzu-Chi University (Approval ID: 104-41, 105-39).

### T cell receptor (TCR) β gene rearrangement clonal typing by PCR

Genomic DNA was isolated from the tumors, the splenocytes from the spleen, and the tail tissue of the tumor-bearing mice. PCR analysis of the Dβ1-Jβ1 and Dβ2-J2 regions of the *TCR* β gene was performed, with a modification from a previous study 19. Briefly, extracted DNA was amplified by 10-cycle touchdown PCR (30 s at 94 °C, 30s at 65 °C, 2 min at 72 °C), followed by 25-cycle PCR (10 s at 94 °C, 30s at 57 °C, 2 min at 72 °C), with β1 primer pair (Dβ1.1: 5'-GAGGAGCAGCTTATCTGGTG-3' and Jβ1.7: 5'-ACCATGGTCATCCAACACAG-3') or β2 primer pair (Dβ2.1: 5'-TAGGCAACCTGTGGGGAAGAAAC-3' and Jβ2.7: 5'-TGAGAGCTGTCTCCTACTATC-3') by using Biometra T1 Thermocycler. After 25-cycles of PCR, the amplified products were resolved on 2% agarose gel, and images were taken by UVP ChemStudio v8.

### HE and IHC staining of tumor

Tumors were dissected, weighed, and subjected to the paraffin section and stained with hematoxylin and eosin (HE). To further identify tumor category, immunohistochemical (IHC) analysis with antibodies against CD20 (1:200 dilution)(sc-7735, Santa Cruz), CD3 (1:200 dilution) (sc-20047, Santa Cruz), pan-cytokeratin (1:100 dilution) (sc-15367, Santa Cruz), IGF1R (1:200 dilution) (sc-462, Santa Cruz), cMET (1:200 dilution) (bs-0668R, BIOSS), and ERα (1:200 dilution) (#8644, Cell Signaling).

### Data analysis

The number of each mouse experimental injection group was at least higher than 6. HE and IHC pictures were presented in one of three independent stainings. Statistical analysis was carried out using Prism Software (GraphPad) and Microsoft Office Excel 2010. For statistical comparison in two independent groups, the data were analyzed using the student's t-test. The log-rank test was to the comparison of the Kaplan-Meier curve. Significant differences were defined as P < 0.05.

## Results

### Injection of human preovulatory follicular fluid induces early-onset lymphoma preferentially at mammary adipose sites in *Trp53 ^-/-^* mice

The FF aspirates were procured from women who underwent the IVF program. Characteristics of the molecular content of human FFs, including the individual and global proteomic profiles, have been well-documented 20, 21. Through the gene ontology analysis of the proteins identified in human FF, the molecular function of FF molecules, including catalytic and binding activity (which presents in 31% of the molecules), metabolic process (19%), cellular process (14%), cell communication (11%), and immune system process (11%) 21. Under this established knowledge of FF, we discovered high levels of IGF2 (287.4 ± 88 ng/ml) and HGF (58.5 ± 20 ng/ml) in 16 FF aspirates. The individual levels IGF2, HGF, and clinic parameters in the 16 FF aspirates were listed in Table 1.

**Figure 1:**
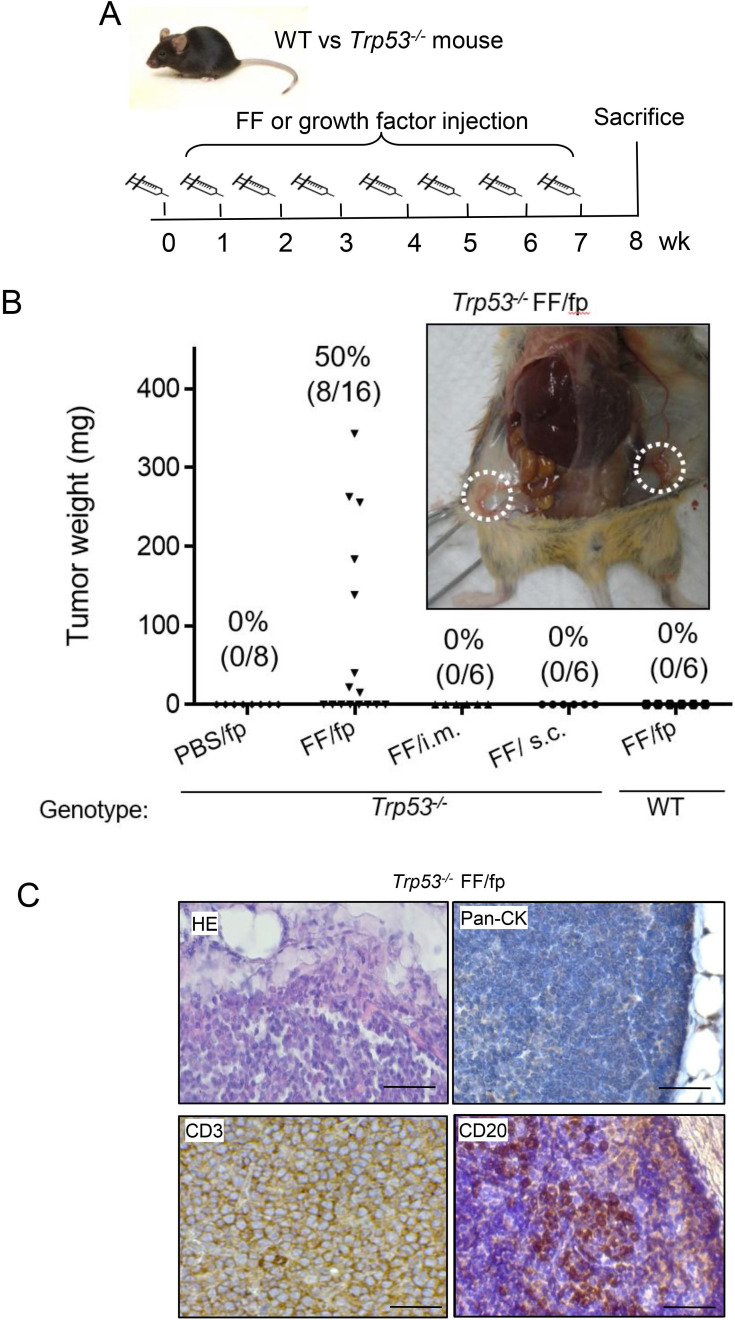
** Tissue-specific tumorigenesis in *Trp53^-/-^* mice induced by human preovulatory follicular fluid injection. (A)** The injection protocol for tumorigenesis to female *Trp53^-/-^* or wild type (WT) mice. FF was injected into the groin mammary fat pad (fp), muscle (i.m.), or subcutaneous tissue (s.c) once a week for up to seven weeks to compare the tumorigenic of different subcutaneous sites in *Trp53^-/-^* mice. PBS fp injection (PBS/fp) as a injection control for FF fp injection (FF/fp) in *Trp53^-/-^* mice, WT mouse with FF fp injection (WT FF/fp) was used as genotype control for p53-null mice with FF fp injection (*Trp53^-/-^* FF/fp) to compare the differences between the two genotypes. Animals were sacrificed at the eighth week. **(B)** Weight and tumor incidence (%) of each tumor in different FF-injection groups and gross tumors at bilateral subiliac lymph node (circled) in a representative FF fp-injected *Trp53^-/-^* mouse are shown. **(C)** HE and IHC stains of a representative FF/fp injection tumor in *Trp53^-/-^* mouse showing pan-cytokeratin (-), CD3(+), CD20 (-) T cell lymphoma (brown). Scale bar: 20 μm.

To investigate the tissue specificity of FF-induced tumorigenesis, we conducted weekly FF injections to different sites of *Trp53^-/-^* mice for up to 7 weeks (Fig. 1A). As shown in Fig. 1B, injections to the mammary fat pat grew tumors in 8/16 (50%) mice. Other injections including subcutaneous, and intramuscular ones did not raise any tumor. In the groin mammary fat pad injections, tumors were mainly found at the adjacent subiliac lymph nodes and spread along the lymphatic drainage nearby the fat depots (Fig. 1B, Supplementary Fig. 1). The HE stain showed the tumor cells were not in epithelial morphology but with round shape, as the type of hematopoietic cells (Fig. 1C). These tumors were diffusely infiltrated by monotonous and round lymphoid cells and there was no histologically normal architecture of the lymph node in the tumor. IHC stain showed these cells were negative of the epithelial pan-cytokeratin marker but universally positive of CD3, and only scattered regions of the tumor having CD20-positive cells which may reflect infiltration with B cells. These histological findings were consistent with T cell lymphoma.

### IGF2 and HGF are largely responsible for the FF-induced tumorigenesis, and can also induce lymphoma by direct injections

**Figure 2:**
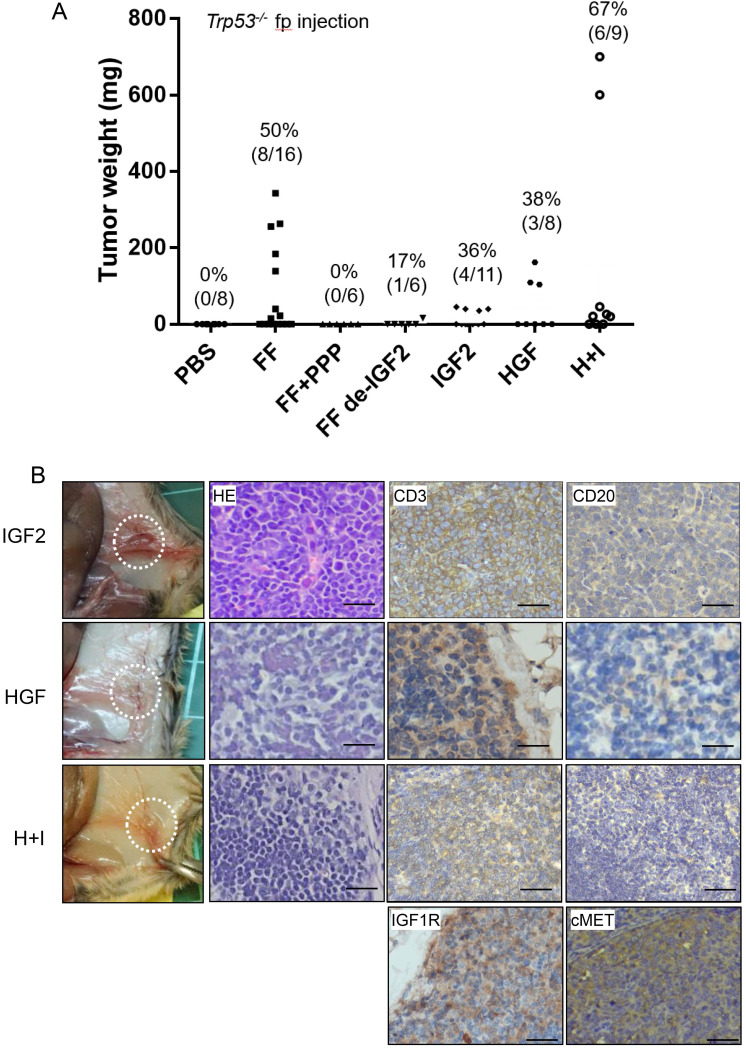
** IGF2 and HGF are responsible for the FF-induced tumorigenesis in *Trp53^-/-^* mice by direct injections. (A)** The weight distribution and incidence (%) of tumors derived from 7 weeks fp injection protocol with FF with or without IGF2 depletion (FF de-IGF2), IGF-1R inhibitor (PPP, 100 nM) co-injection, and with recombinant human IGF2 (100 ng/ml), HGF (20 ng/ml) and their combination (H+I). **(B)** Representative pictures show the gross appearance (circled), HE stain, and IHC stain for CD3 (brown), CD20 (-), IGF1R and cMET (brown) of the lymphoma. Scale bar: 20 μm.

In previous studies, we have discovered the IGF2 axis proteins in FF confers stem cell activation and transformation activities on fallopian tube fimbrial epithelium (FTE) 17. We also discovered a high level of HGF in FF, and could also transform FTE cells independently (unpublished results). We tested whether these two growth factors are responsible for the lymphomagenic activity of FF. Tumorigenesis was completely inhibited in six mice when IGF-1R inhibitor picropodophyllin (PPP) was injected together with FF. To clarify how much does IGF2 contribute to the tumorigenesis activity in FF, we depleted IGF2 from FF by neutralizing monoclonal antibody. After IGF2 depletion, the tumorigenic rate reduced to 17% (1/6) (Fig. 2A). Also, replacing FF with pure IGF2 at 100 ng/ml could induce tumors in 4 of 11 (36%) mice. Similarly, injection of pure HGF at 20 ng/ml, induced tumors in 3 of 8 (38%) mice. When HGF and IGF2 were co-injected, 6 of the 9 (67%) mice developed tumors. These IGF2 and HGF-induced tumors showed the same anatomic distribution and histology of CD3(+)/CD20(-) T cell lymphoma.

We also performed immunohistochemistry on tumor tissue sections for the receptors, IGF1R, and cMET, and confirmed their expression (Fig. 2B). Meanwhile, many studies have shown the expression of IGF1R and cMET in lymphomas, which regulate tumor growth and promote malignant transformation 22-24.

### Transplantation and rearrangement of the TCRβ gene provide evidence of the T cell lymphomatosis

**Figure 3:**
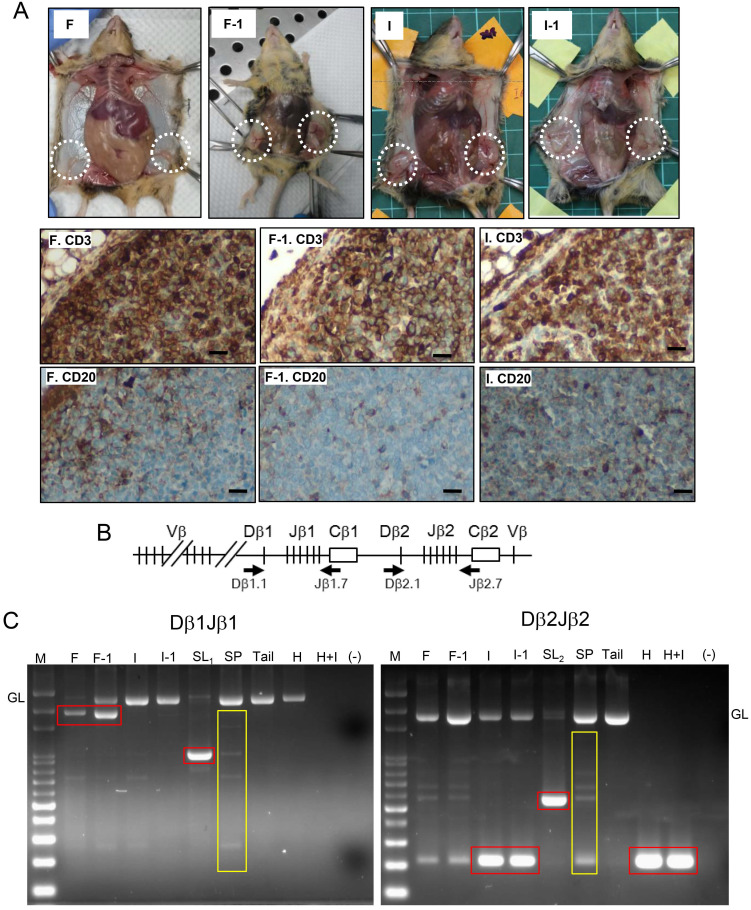
** Verification of T cell lymphoma by showing tumorigenesis in serial transplantation and monoclonal TCRβ gene rearrangement. (A)** Gross tumorigenesis and CD3(+)/CD20(-) expression pattern (brown) in tumors derived from fat pad injection with FF (F) or IGF2 (I), and in their daughter tumors retrieved 2 weeks after serial transplantation (F-1, I-1, n=3 in each group). Scar bar: 20 μm. **(B)** Schematic diagram of the *TCRβ* gene locus. Locations of the two primer sets used for PCR are shown (Arrows). **(C)** Dβ1Jβ1 and Dβ2Jβ2 rearrangements was examined in three representative lymphomas induced by FF (F), IGF2 (I), HGF (H), HGF+IGF2 (H+I), their transplantation offsprings (F-1, I-1) as well as two spontaneous lymphomas developed at old age (SL_1,2_). Isolated splenocytes (SP) and tail tissue served as polyclonal TCR rearrangements and non-rearranged germline (GL) controls, respectively. Rearranged TCRβ fragments in the tumor (monoclonal) are boxed in red, those of background lymphocyte infiltrations or the control splenocytes (polyclonal) are boxed in yellow.

Given that the nonspecific inflammation or lymphoproliferative disordered maybe both induced by FF which contains high-level inflammatory cytokines 25, we further detected the tumorigenesis by serial transplantation and tumor monoclonality by analyzing the clonal status of the rearranged *TCRβ* gene. As shown in Fig. 3A, both the FF-induced tumor and IGF2-induced tumor, when transplanted to a new recipient, rapidly grew the same CD3(+)/CD20(-) tumor after two weeks. PCR analysis of clonal rearrangement of the *TCRβ* gene locus (Fig 3B) demonstrated that all four (FF, IGF2, HGF, HGF+IGF2) induced lymphomas, like the spontaneous lymphomas grown at old age, had a prominent monoclonal Dβ-Jβ rearrangements. This is in contrast to the polyclonal nature of splenocytes as well as the background infiltrating lymphocytes in the tumor. Specifically, the FF-induced lymphoma, its transplant, and a spontaneous lymphoma showed Dβ1Jβ1 recombination (Fig. 3C, left), whereas the IGF2-induced lymphoma, its transplant, HGF-induced lymphoma, IGF2+HGF-induced lymphoma, and another spontaneous lymphoma showed Dβ2J2 rearrangement (Fig. 3C, right). Overall, these results provide evidence that our model can promote lymphomagenesis in female *Trp53^-/-^* mice.

### Estrogen/ER signaling is required for the FF-induced lymphomagenesis

**Figure 4:**
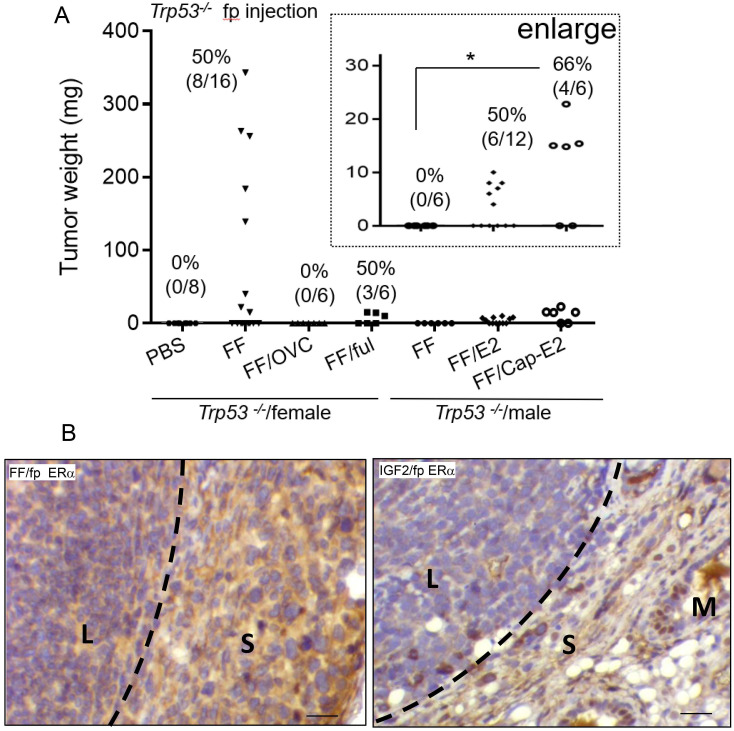
** Estrogen/ERα signaling is largely required for the growth of lymphoma. (A)** The weight distribution and incidence (%) of tumors derived following the 7 weeks fp injection protocol, FF was injected into the mammary fat pad of female *Trp53^-/-^* mice with or without ovariectomy (OVC) or co-injection with Fulvestrant (5mg per mouse) (FF/ful). The same FF injection was also tested in male *Trp53^-/-^* mice, with or without co-injection with 80 nM estradiol (E2), or in mice implanted with E2-capsule (80 nM) (Cap-E2). **(B)** IHC stain of ERα (brown) is shown in the representative tumor tissue induced by FF mammary fat pad injection (FF/fp) or IGF2 mammary fat pad injection (IGF2/fp) injection. Locations of the lymphoma (L), and adjacent stroma (S) and mammary gland (M) are shown. Scale bar 20 μm. * p<0.05 by student t-test.

Since the mammary fat pad is an estrogen-dependent tissue conferring mammary gland development 26, and FF typically harbors an extremely high level of E2, we investigated the role of estrogen signaling in this tumorigenesis model. Compared to the high tumorigenic rate in the female *Trp53^-/-^* mice after FF injection, the same injection did not result in tumorigenesis in male mice (0/6) or female mice after ovariectomy (0/6) (Fig. 4A). When FF was co-injected with a fulvestrant, an ER receptor antagonist, we observed tumorigenesis in 3/6 female mice, all of which were small tumors localized at the injection site. The mean weight was 13.2 ± 2.8 mg, as compared to 158 ± 125 mg without fulvestrant co-injection (Fig. 4A). With E2 supplement, either by co-injected with FF or by the implant of slowly releasing capsule, male *Trp53^-/-^* mice gave rise to local tumorigenesis in 6/12 (50%) and 4/6 (66%) of mice, respectively (Fig. 4A). Again, tumors in these two groups were small, with a mean weight of 7.2 ± 2 and 17 ± 3.9 mg respectively. Notably, in the capsule-releasing E2 group, the tumor growth level had statistically significant compared to the group without E2 (Fig. 4A, enlarged region). We further examined the ERα expression in these tumors induced by FF or IGF2 injections. ERα was expressed only in the stroma surrounding the lymphoma and adjacent adipose tissue stroma, but not in the lymphoma cells (Fig. 4B). These results suggest an estrogen-dependent lymphomagenesis, acting through the microenvironment.

### Extended exposure to FF does not increase the tumorigenic rate

**Figure 5:**
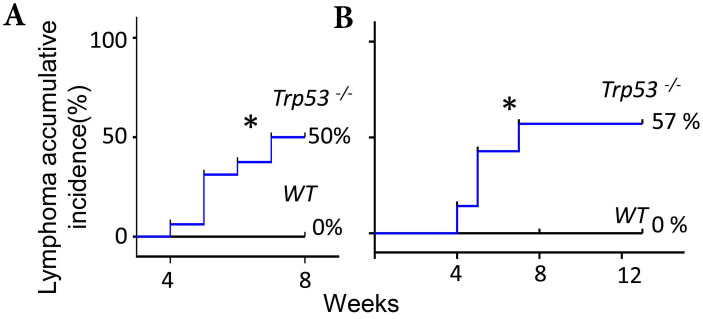
** Extended exposure to FF does not increase the tumorigenic rate. (A)** The Kaplan-Meier curve shows lymphoma accumulative incidence of lymphomagenesis of 16 *Trp53^-/-^* and 8 WT mice by FF in the 7 weeks mammary fat pad injection protocol. **(B)** Another 7 *Trp53^-/-^* and 7 WT mice were tested by extending injection, in which the fad pad injection by FF was up to 13 weeks, and lymphoma accumulative incidence of lymphomagenesis is shown. * p<0.05 by Log-rank test to Kaplan-Meier curve.

The FF- or IGF2/HGF-induced lymphomagenesis in this *Trp53^-/-^* mouse model seems to be stochastic because only part of the injected mice developed a tumor. FF could induce accumulative incidence of lymphoma in 8/16 (50%) of mice by seven weeks (Fig. 5, Fig. 1B), we wondered whether an extended injection protocol would confer a higher incidence of tumorigenesis. Upon extending the weekly injection from 7 weeks to 13 weeks in another experimental cohort, the lymphoma incidence remained at 57% (4/7) at the 8^th^ and 14^th^ week, which was similar to the incidence of 50% (8/16) in the 7 weeks injections (Fig. 5). Meanwhile, a similar 59% (33/56) tumorigenic rate was found in a study observing the spontaneously developed lymphoma in the same p53-null strain 27. In this spontaneous lymphomagenic model, the tumor was observed at age of 5 months 12, 27, which was older than the age we observed tumors. This result is compatible with a clonal expansion effect of FF or IGF2/HGF on the pre-initiated cancer cells under the evolution at a null *Trp53*.

Augmented by the extended injection, the lymphoma developed diffusely over the subcutaneous lymph nodes, the subiliac lymph node near the injection site, the axillary node, the superficial parotid, and submandibular nodes (Supplementary Fig. 1A, B). These mice with advanced lymphoma also had severe splenomegaly with a fuzzy spleen medulla zone indicating severe inflammation in histopathology (Supplementary Fig. 1C).

## Discussion

Lymphomas develop spontaneously in *Trp53^-/-^* mice with C57BL/6 background typically at a mean age of about 5 months 27 12. In the same *Trp53^-/-^* mice, we found a weekly injection of human FF, pure IGF2, HGF, or their combination to the mammary fat pad induced tumorigenesis by age of 15 weeks, which was earlier than the spontaneous model 12. In contrast to other human cancers which mostly happening at old ages, lymphoma tends to occur in all age groups and is the most common cancer in young people. The etiology of early-onset lymphoma is elusive. Earlier studies have shown a decreased risk in association with higher birth order, multiple siblings, crowded living conditions, exposure to daycare/kindergarten, and lower socioeconomic status 28-31. Thus an early exposure hypothesis proposes that either a deficit of fecal-oral exposures or a delayed infection with the specific pathogen in early life may disrupt the Th1/Th2 immune response milieu and relates to the development of lymphoma 32. However, the molecular mechanism causing the early onset remains unknown. This study, for the first time, demonstrated that the stem cell clonal expansion factor IGF2 and mitogenic factor HGF confer an early onset of T-cell lymphoma in *Trp53^-/-^* mice. Thus, the combination of *Trp53* driver mutation, genomic instability, and clonal expansion of initiated stem cells by IGF2 and HGF may be an important mechanism of lymphomagenesis.

For the first time, the study found that weekly injection of IGF2, HGF, and their combination in Trp53-null mice could directly induce lymphomas in 36%, 38%, and 67% of mice, respectively. The results demonstrated an efficient and straightforward mouse lymphomagenesis model. By weekly injection of pure IGF2 and HGF into the mammary fat pad in p53-null mice, tumorigenesis could be achieved by seven weeks in two-third of the mice.

IGF2 is the growth hormone specifically for the development of the fetus and ovarian follicle 33-35. *IGF2* is also a maternally imprinted gene that is commonly overexpressed in cancer cells through loss of imprinting (LOI) 36. IGF2 exerts its effects by binding to the IGF-1 receptor (IGF-1R) via the AKT-mTOR, and AKT-OCT4-NANOG signaling pathways. In the ovulation-induced transformation model, FF-IGF2, under these two signaling pathways, readily induces stemness, spheroid formation, and clonal expansion of oviductal epithelial cells that have been initiated by loss of function of p53/Rb and DNA aneuploidy upon cell passage 17. A recent study also showed that IGF1, the adult-type IGF sharing the same IGF axis signaling with IGF2, is responsible for the initiation of lung cancer recurrence by inducing self-renewal and clonal expansion of cancer stem cells and results in subsequent neoangiogenesis and recurrence 37. Meanwhile, the serum level of IGF-1 is an important indicator of the risk of occurrence of hematopoietic cancers including lymphoma 38, 39.

The present study presents the first finding that estrogen/ER signaling contributes to lymphomagenesis in Trp53 knocked out mice. To validate the estrogen/ER-dependence in the FF-induced lymphomagenesis, we negatively confirmed the finding by showing compromised tumorigenesis in female ovariectomized mice, mice with ER antagonist treatment, and in male mice. Besides, positive validation was showed by gaining tumorigenesis in male mice after two different ways of E2 supplement. The estrogen/ER dependent tumorigenesis in this IGF2/HGF and Trp53-null model of lymphomagenesis may suggest a gender difference of this disease. In a sophisticated transgenic study of the effect of doses of *Igf2* in the *Trp53* gene on tumorigenesis, Haley *et al*. found that conditional homozygous deletion of *Igf2* significantly delays the onset of the p53-null tumor phenotype. In contrast, biallelic expression of *Igf2* accelerated tumor formation in *Trp53* heterozygous animals. Interestingly, the tumor promotion effect of biallelic *Igf2* expression only happened in female mice 40. By revealing E2/ER is required for the tumorigenic effect of FF-IGF2, the present study may disclose the mechanism of this gender difference. Accordingly, lymphomagenesis was observed only in female mice but not in male mice unless estrogen was supplemented. Inhibition of E2/ER by ovariectomy, ER antagonist in female mice also largely disrupted the activity. Thus, in the context of p53 loss, IGF2 might enhance the tumorigenicity depending on E2 and ER.

FF has a complex composition rich in hormones, mitogens, mutagen, and inflammatory cytokines and chemokines 16, 25. Characteristics of the molecular content of human FFs, including the individual and global proteomic profiles, have been well-documented 20, 21. Through the gene ontology analysis of the proteins identified in human FF, the molecular function of FF molecules, including catalytic and binding activity (which presents in 31% of the molecules), metabolic process (19%), cellular process (14%), cell communication (11%), and immune system process (11%)^21^ have been displayed. Accordingly, we identified and verified that IGF2 and HGF are the two most important growth factors in FF 17. In this study, we collected 16 FF aspirates and discovered high levels of IGF2 and HGF (Table 1).

We found pure IGF2 in a concentration equivalent to that in FF and the same injection protocol could induce tumors in 36% of the mice. However, after depletion of IGF2, FF still induces tumors in 1/6 mice. This suggests the presence of other transforming agents in FF. Indeed, we found HGF is another transforming factor in FF that induced lymphomagenesis in 38% of the injected mice. In bone marrow hematopoiesis, HGF produced by the marrow stromal cells was found to promote survival, proliferation, and adhesion of CD34+ hematopoietic progenitors which express its receptor c-MET 41. Cumulative studies also disclosed high expression of c-MET and HGF in different types of lymphoma and leukemia and their microenvironment, respectively; or both in the tumor cells. These paracrine and autocrine signalings promote the transformation and progression of the malignancies 22, 42, 43. Meanwhile, we found through c-MET/MAPK and c-MET/AKT pathways, FF-HGF induces proliferation, migration, anchorage-independent growth, and tumorigenesis of immortalized fallopian tube epithelial cells [unpublished results].

The findings of this study also suggest an active role of the adipose microenvironment in lymphomagenesis. Particularly, lymphatic circulation provides a critical route for lipid transport to the blood and fat deposits, allowing for normal fat expansion. This is the reason why the lymph node always exists in adipose tissue. The study showed tumorigenesis specifically when FF was injected into adipose-rich tissue but not in non-adipose tissues such as dorsal subcutaneous, and muscle. The CD3-positive lymphocytes were frequently present in adipose-rich tissues but not in the muscular or the subcutaneous. This may explain the adipose tissue-specific efficacy of this injection model. The other possibility, which reveals to be more likely, is an active role of adipose microenvironment in lymphomagenesis. Many pre-clinical and clinical studies have linked adiposity with the increased occurrence, progression, and metastasis of different cancers 44. Cancer-associated adipocytes are known to promote invasion and metastasis of cancer and provide energy to fuel tumor growth 45-47. Particularly in the case of mammary carcinogenesis, the mammary adipose tissue has been shown to promote tumor growth and metastasis in an estrogen-dependent manner 48.

## Conclusion

**Figure 6:**
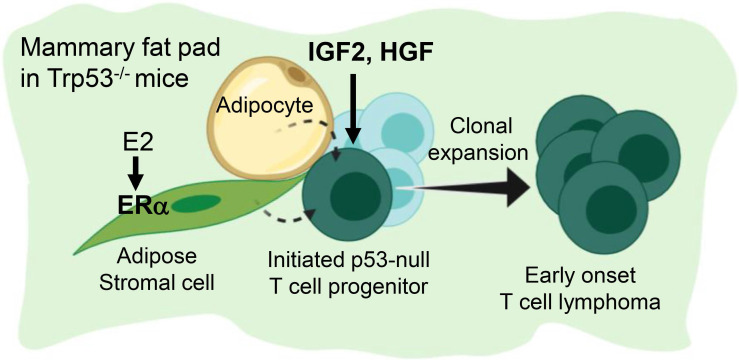
** Estrogen- and adipocyte-dependent lymphomagenesis augmented by IG2 and HGF in Trp53-null mice.** In the background of a loss of Trp53, initiated tumor cell clones are expanded by IGF2 and HGF to develop into early-onset lymphomas. This lymphomagenesis happens specifically in the adipose microenvironment and depends on the E2/ERα signaling.

Figure 6 summarize the finding of this study. Under a background of a loss of p53, initiated tumor cell clones are expanded by IGF2/IGF1R and HGF/cMET signals to develop into early-onset lymphomas. This lymphomagenesis happens preferentially in the adipose microenvironment where the E2/ERα signaling is required. The study established an efficient lymphomagenic model by fat pad injection of IGF2 and HGF in p53-null mice. The new model yields gross tumors in the mammary fat pad in a shorter time. About half of the injected mice developed tumors by 7 weeks of induction. In addition to the study of p53 driver mutation in the development of lymphoma, the interesting estrogen dependence and sex dimorphism of the tumorigenic effect, as well as the protumorigenic propensity of the adipose microenvironment, all add to new dimensions of research on this lymphomagenic mouse model.

## Supplementary Material

Supplementary figure S1.

## Figures and Tables

**Table 1:** IGF2, HGF levels in FF collected from 16 women undertaking IVF program

ID	Age	Etiology of infertility	Controlled ovarian hyper-stimulation protocol	IGF2 level (ng/ml)	HGF level (ng/ml)	Fertilization rate (%)	No. of ET	Clinical outcome
140070	32	Male factor	Long protocol GnRH analog with rFSH	351	66.8	95%	3	Born singlet
140099	39	Anovulation	CC + Menopur + Gonal-f + Cetrotide	109	94.8	100%	3	No pregnancy
140100	32	Repeat IUI failure	Elonva + Cetrotide + Gonal-f	214.6	66.0	63%	3	No pregnancy
140117	35	Tubal factor	Triptorelin acetate + Gonal-f	348	72.6	100%	0	No pregnancy
140125	23	Anovulation	Elonva + Cetrotide + Gonal-f	237	75.0	70%	0	No pregnancy
140134	36	Repeat IUI failure	CC + Elonva + Gonal-f+ Lupron	297	71.6	100%	3	No pregnancy
140135	33	Male factor	Elonva + Gonal-f + Cetrotide	127.5	70.5	69%	3	Born singlet
140139	42	Tubal factor	Lupron + Elonva + Puregon	293.9	81.2	100%	3	No pregnancy
140143	47	Anovulation	CC + Menopur + Gonal-f	279.4	61.5	100%	0	No pregnancy
150028	37	Tubal factor	Lupro + Gonal-f	365	31.1	80%	3	No pregnancy
150098	28	Repeat IUI failure	CC + Gonal-f + Cetrotide + Lupro + Ovidrel	408	47.4	100%	1	No pregnancy
150102	36	Tubal factor	Puregon + Cetrotide + Lupro + Ovidrel	266	34.9	100%	4	Missed abortion
150113	32	Male factor	Lupro + CC + Puregon + HCG	363.4	28.1	75%	0	No pregnancy
150121	42	Male factor	Lupro + CC + Gonal-f	287.5	43.4	25%	1	No pregnancy
150127	42	Male factor	CC + Gonal-f + Cetrotide + Menopur + HCG + Ovidrel	407.8	57.3	29%	2	No pregnancy
150135	43	Repeat IUI failure	CC + Gonal-f + Saizen + Cetrotide	243.6	33.0	0	0	No pregnancy

CC: Clomiphene citrate, GnRH: Gonadotropin releasing hormone, rFSH: reombinant follicular stimulating hormone, Menopur: FSH+LH, Gonal-f: Follitropin alpha, Cetrotide: Cetrorelix acetate, Elonva: Corifollitropin alfa, Lupron: Leuprolide acetate, Puregon: Follitropin beta, Ovidrel: Choriogonadotropin alfa, Saizen: Somatropin, ET: embryo transfer.

## Abbreviations

FF: follicular fluid
IGF2: Insulin-Like Growth Factor-2
HGF: Hepatocyte Growth Factor
E2: estradiol
ER: estrogen receptor
IVF: *in-vitro* fertilization
HE: hematoxylin and eosin
IHC: immunohistochemical
FTE: fallopian tube fimbrial epithelium
PPP: picropodophyllin
