# Supplementary material for: Insuline-Like Growth Factor-2 (IGF2) and Hepatocyte Growth Factor (HGF) Promote Lymphomagenesis in p53-null Mice in Tissue-specific and Estrogen-signaling Dependent Manners
Source: J Cancer. 2021 Aug 21;12(20):6021–30. doi: 10.7150/jca.60120 (PMC8425200; doi:10.7150/jca.60120)

Supplementary Fig 1.

(A) The extended FF injection protocol to the groin mammary fat pad of *Trp53*$^{-/-}$ mice for up to 13 weeks. (B) A representative dissection shows the spreading of lymphoma from the subiliac node (1) to the axillary node (2) and the superficial parotid and submandibular nodes (3). (C) Gross appearance and histology of spleens showing splenomegaly.

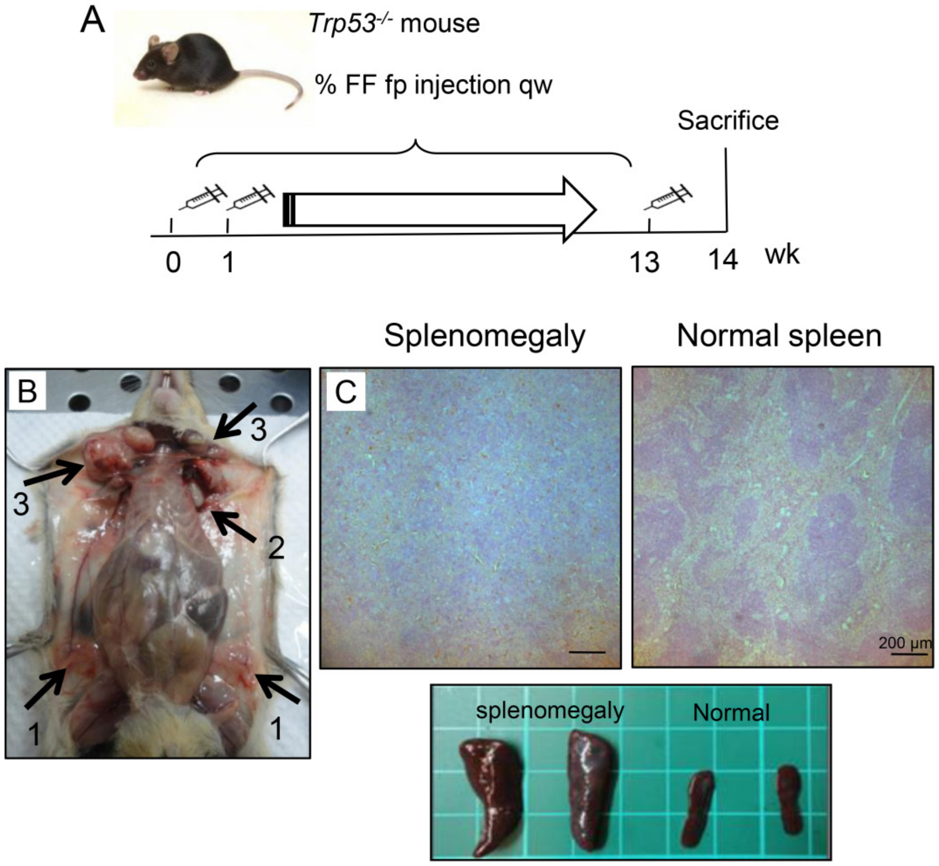

Supplement: Supplementary file 1 — Supplementary figure S1. [file jcav12p6021s1.pdf]
